# Supplementary material for: MetaRibo-Seq measures translation in microbiomes
Source: Nat Commun. 2020 Jun 29;11:3268. doi: 10.1038/s41467-020-17081-z (PMC7324362; doi:10.1038/s41467-020-17081-z)
Supplement: Supplementary file 10 — Supplementary Data 7 [file 41467_2020_17081_MOESM10_ESM.zip › File2/Confidence_High_Taxonomy/148132_out.krona.html]

Javascript must be enabled to view this page.

members
magnitude
magnitudeUnassigned
count
unassigned
taxon
rank

148132\_out

3

2
3
superkingdom

1239
phylum
3

3
class
186801

3
order
186802

3
family
541000

3
genus
216851

1971605

SRS104400\_contig\_number\_44493SRS144506\_contig\_number\_45198SRS149075\_contig\_number\_14716
3
species
